# Resuscitation of Ischemic Donor Livers with Normothermic Machine Perfusion: A Metabolic Flux Analysis of Treatment in Rats

**DOI:** 10.1371/journal.pone.0069758

**Published:** 2013-07-26

**Authors:** Maria-Louisa Izamis, Herman Tolboom, Basak Uygun, Francois Berthiaume, Martin L. Yarmush, Korkut Uygun

**Affiliations:** 1 Center for Engineering in Medicine, Massachusetts General Hospital, Harvard Medical School, and the Shriners Hospitals for Children, Boston, Massachusetts, United States of America; 2 Division of Cardiac and Vascular Surgery, University Hospital Zurich, Zurich, Switzerland; 3 Department of Biomedical Engineering, Rutgers University, Piscataway, New Jersey, United States of America; Rutgers University, United States of America

## Abstract

Normothermic machine perfusion has previously been demonstrated to restore damaged warm ischemic livers to transplantable condition in animal models. However, the mechanisms of recovery are unclear, preventing rational optimization of perfusion systems and slowing clinical translation of machine perfusion. In this study, organ recovery time and major perfusate shortcomings were evaluated using a comprehensive metabolic analysis of organ function in perfusion prior to successful transplantation. Two groups, Fresh livers and livers subjected to 1 hr of warm ischemia (WI) received perfusion for a total preservation time of 6 hrs, followed by successful transplantation. 24 metabolic fluxes were directly measured and 38 stoichiometrically-related fluxes were estimated via a mass balance model of the major pathways of energy metabolism. This analysis revealed stable metabolism in Fresh livers throughout perfusion while identifying two distinct metabolic states in WI livers, separated at t = 2 hrs, coinciding with recovery of oxygen uptake rates to Fresh liver values. This finding strongly suggests successful organ resuscitation within 2 hrs of perfusion. Overall perfused livers regulated metabolism of perfusate substrates according to their metabolic needs, despite supraphysiological levels of some metabolites. This study establishes the first integrative metabolic basis for the dynamics of recovery during perfusion treatment of marginal livers. Our initial findings support enhanced oxygen delivery for both timely recovery and long-term sustenance. These results are expected to lead the optimization of the treatment protocols and perfusion media from a metabolic perspective, facilitating translation to clinical use.

## Introduction

Transplantation is currently the only treatment option for end-stage liver disease but it is limited by the lack of high quality donor organs. An area of keen investigation is into the use of machine perfusion (MP) as a means of resuscitating presently non-transplantable donor organs after cardiac death (DCD), thereby significantly increasing the donor organ pool [1], [2]. Porcine and murine models of DCD livers are significantly improved by MP compared to organs preserved by the gold standard of static cold storage [3], and furthermore, normothermic machine perfusion (NMP) is necessary for their successful transplantation [4], [5], [6], [7].

While MP is particularly well-suited to dynamic evaluation of organ function, methods for standardized quantitative analysis are only emerging very recently [8]. Optimization of perfusion parameters such as temperature, flow rate, perfusion media and the ideal oxygen carrier [9] continue to dominate most research efforts. Two fundamental questions remain unanswered: What should the liver be perfused with and how long should it be perfused for?

Optimizing the perfusate addresses the optimization of the metabolic state of the liver during MP. During perfusion, ATP levels recover [10], [11] alleviating some aspects of ischemia-reperfusion injury, and this is well-correlated with enhanced transplant success. However, existing studies typically focus on a few global indicators of energy recovery, which by themselves are nonspecific, and so do not enable definitive conclusions to be reached regarding the limitations of the perfusion system and protocol employed. Lactate accumulation, for example, occurs during anaerobic metabolism from hypo-perfusion [12], normal erythrocyte metabolism [13] and in the absence of an initial lactate concentration in the perfusion medium [14]. Other frequently used parameters, such as trends in glucose metabolism, degree of oxygen consumption, ability to produce albumin or other complex proteins, rates of excess amino acid breakdown and urea formation [3], [15], [16] similarly reflect multiple likely pathways of activity that will provide more specific information if evaluated in a cohesive manner.

Endeavoring towards a standardized approach to organ recovery and rational perfusion optimization, we employed a comprehensive Metabolic Flux Analysis [17] model of whole-organ metabolism [18] to dynamically assess the function of the liver during perfusion. The mathematical model stoichiometrically correlates 28 measured and 34 calculated metabolite rates of uptake or release (i.e. fluxes) from hourly perfusion time points of organs that were subsequently successfully transplanted (>1 month survival). Using the temporal profiles of the measured fluxes, a significant metabolic turnaround signaling recovery of warm ischemic livers, was observed within 2 hours of perfusion; oxygen uptake rate (OUR) most dramatically illustrated this event. Metabolic Flux Analysis (MFA) comparison of perfused ischemic livers to perfused Fresh livers highlighted the impact of NMP on organ function and identified system-specific shortcomings. This approach enables future perfusion optimizations for long-term organ storage and recovery.

## Materials and Methods

### Animals

Male Lewis rats weighing 200–300 g were obtained from Charles River Laboratories (Wilmington, MA) and maintained in accordance with National Research Council guidelines. The Subcommittee on Research Animal Care, Committee on Research at the Massachusetts General Hospital, approved the experimental protocols. All animals were allowed to acclimatize for at least 2 days prior to any experimentation. Procured livers were either Fresh (n = 11) or exposed to an hour of warm ischemia (WI, n = 7).

### Isolation of Donor Livers

Full details of the procedure are described elsewhere [5]. Briefly, a transverse abdominal incision was made and the intestines retracted to expose the portal vein (PV), the common bile duct (CBD), and the inferior vena cava (IVC). The CBD was cannulated (22 G polyethylene stent, Surflo, Terumo, Somerset, NJ) and the IVC freed from the right renal and adrenal veins. The portal vein (PV) was freed from the splenic and gastroduodenal veins. The right phrenic vein emptying into the supra-hepatic vena cava (SHVC) was ligated. Heparin (200 U) was injected into the penile vein. The IVC and PV were clamped. For Fresh livers, an 18G polyethylene cannula (Terumo) was inserted into the PV, and the liver was flushed with 5 mL of cold (4°C) University of Wisconsin (UW) solution (Viaspan, Barr Laboratories, Pamona, NY). The diaphragm was opened, the SHVC was transected, and the liver was flushed with an additional 5 mL of UW solution. The liver was removed, weighed, and placed in a bowl of ice-cold UW solution to be cuffed. For WI livers, flushing of the organ via the PV was omitted. After isolation from the donor, these livers were then weighed and placed in a temperature-controlled chamber filled with saline and maintained at 34±0.1°C for 1 hr. During this period the portal vein (PV) and inferior vena cava (IVC) were cuffed.

### Normothermic Liver Perfusion and Metabolite Sampling

The perfusion medium comprised phenol red-free Williams Medium E (WE, Sigma Chemical, St. Louis, MO) chosen for its similarity to *in vivo* influxes (Appendix S1 and Table 1). WE was supplemented with 2 u/L insulin (28.85units/mg Humulin, Eli Lily, Indianapolis, IN), 100,000 u/L penicillin, 100 mg/L streptomycin sulfate (Gibco, Invitrogen, Grand Island, NY), 0.292 g/L L-glutamine (Gibco), 10 mg/L hydrocortisone (Solu-Cortef, Pharmacia & Upjohn, Kalamazoo, MI), and 1000 u/L heparin (APP, Schaumberg, IL). The primary circuit of the perfusion system comprised perfusion medium (“perfusate”) that recirculated by means of a peristaltic pump through a jacketed perfusion chamber, a membrane oxygenator, a heat exchanger, and a bubble trap. The oxygenator was gassed with a mixture of 74%N_2_/21%O_2_/5%CO_2_ and 100%O_2_ to maintain a constant pH. Fresh rat plasma (25% v/v) and erythrocytes (18–20% v/v) were collected earlier [5] and added to the perfusate. The total perfusate volume was 55–60 mL. Perfusate hematocrit was sustained, nutrients were replenished, and metabolism by-products were diluted through dialysis. A hollow fiber dialyzer with a 2200 cm^2^ membrane area and a 30 kDa nominal molecular cutoff weight (SpectrumLabs, Rancho Dominguez, CA) enabled counter-current mixing of perfusate in the primary circuit with a reservoir of WE (“dialysate”) in a secondary circuit. Temperature within the system was maintained at 37.5°C. Upon completion of cuffing of Fresh livers (∼5 min) and after the period of warm ischemia for WI livers, they were immersed in perfusate in the perfusion chamber. Livers were perfused at a constant flow rate through the portal vein [5] while maintaining portal pressure between 10–12 cmH_2_O. The effluent flowed freely from the SHVC and IVC into the surrounding medium. Perfusate and dialysate samples (1 mL) were collected hourly from the liver effluent and reservoir respectively. Perfusate samples were first spun down at 3000 g before storing the supernatant at −80°C. Oxygen delivery and exit rates were determined from samples taken immediately prior to entry to the portal vein and from the IVC. When the recipient hepatectomy was prepared, the liver was disconnected from the circuit, rinsed in a bowl of saline at room temperature, and weighed again before transplantation.

**Table 1:** Measured Influx Values.

Metabolite Influx	*In vivo* * (PV+HA)	Perfusion**
Albumin (g/min/g liver)	0.02–0.05	0.00
Lactate (mmol/min/g liver)	0.03–0.1	0.00
Glucose (mg/min/g liver)	1.13–2.5	3.68
Alanine (umol/min/g liver)	0.36–0.92	1.86
Ammonia (umol/min/g liver)	0.11–0.2	0.00
Arginine (umol/min/g liver)	0–0.5	0.53
Asparagine (umol/min/g liver)	0.04–0.12	0.28
Aspartate (umol/min/g liver)	0.02–0.04	0.41
Cysteine (umol/min/g liver)	0.01–0.03	0.61
Glutamate (umol/min/g liver)	0.07–0.15	0.56
Glutamine (umol/min/g liver)	0.28–0.68	3.64
Glycine (umol/min/g liver)	0.26–0.6	1.23
Histidine (umol/min/g liver)	0.1–0.35	0.18
Isoleucine (umol/min/g liver)	0.07–0.23	0.70
Leucine (umol/min/g liver)	0.21–0.66	1.05
Lysine (umol/min/g liver)	0.18–0.54	1.10
Methionine (umol/min/g liver)	0.04–0.1	0.18
Ornithine (umol/min/g liver)	0.11–0.28	0.00
Phenylalanine (umol/min/g liver)	0.05–0.13	0.28
Proline (umol/min/g liver)	0.16–0.36	0.48
Serine (umol/min/g liver)	0.17–0.48	0.18
Threonine (umol/min/g liver)	0.19–0.47	0.62
Tyrosine (umol/min/g liver)	0.05–0.17	0.51
Valine (umol/min/g liver)	0.12–0.43	0.79

* *In vivo* influx is the combined portal vein (PV) and hepatic artery (HA) contribution to that flux.

** Perfusate influx is calculated according to the initial perfusate concentrations and a flow rate of 1.8 ml/min/g liver.

#### Biochemical Assays

Blood gases were determined immediately using a blood gas analyzer (Rapidlab, Chiron Diagnostics, Norwood, MA). Oxygen concentration delivered and removed from the liver was calculated using the following equation: [*O_2_*] = (1.39×[*Hb*]×*FO_2_Hb*)+0.00314×*pO_2_*, which expresses the concentration of oxygen (ml/dL of blood) as the sum of oxygen bound to hemoglobin and free in plasma. [Hb] (g/dL) is the concentration of hemoglobin, FO_2_Hb is the fraction of oxyhemoglobin present, 1.39 (mlO_2_/g Hb) is the binding capacity of oxygen to hemoglobin and 0.00314 (mlO_2_/dL/mmHg) is the solubility coefficient of oxygen in plasma, which is dependent on the partial oxygen tension in the blood, pO_2_ (mmHg). The rate of oxygen delivery to the liver (ODR) and exit from the liver (OER) is subsequently a product of the oxygen concentration [O_2_] at the site of entry and departure to the liver, respectively, and the flow rate V (ml/min), normalized to the weight W (g) of the liver. The difference between the two rates provides the hepatic oxygen uptake rate: OUR = ODR-OER.

Similarly, total carbon dioxide release rate (CRR) at each time point was calculated as the difference between total carbon dioxide (tCO_2outlet_) released at the outlet of the liver and the total carbon dioxide entering the liver (tCO_2inlet_), multiplied by the flow rate V (ml/min) and normalized to the weight of the liver, W (g). Total carbon dioxide of perfusate samples taken at the inlet and outlet of the liver was measured via Piccolo Blood Chemistry Analyzer (Abaxis).

Urea was assayed by reaction with diacetyl monoxime using a commercial assay kit (BUN, Sigma-Aldrich, St. Louis, MO). Ketone bodies were measured enzymatically by following the appearance of NADH in the conversion to acetoacetate and the disappearance of NADH in the conversion to β-hydroxybutyrate in the presence of β-hydroxybutyrate dehydrogenase [19]. Nineteen of the common amino acids (except tryptophan) and ammonia were fluorescently labeled using the AccQ-Tag system (Waters Co., Milford, MA), separated by high-performance liquid chromatography (HPLC; Model 2690, Waters Co.) and quantified by a fluorescence detector (Model 474, Waters Co.), as previously described [18]. Lactate was measured using the enzymatic conversion to pyruvate and hydrogen peroxide with lactate oxidase from a commercially available kit (Trinity Biotech, Berkeley Heights, NJ). Albumin concentration was determined by an enzyme-linked immunosorbent assay using a polyclonal antibody to rat albumin [20]. A standard curve was derived using chromatographically purified rat albumin (Cappel Laboratories, Aurora, OH) dissolved in medium. Note that the dialyzer molecular cutoff weight was determined so that albumin could not pass through, and subsequently did not appear in the secondary dialysate circuit. Glucose measurements were quantified with an enzymatic assay kit through conversion to 6-phospho-gluconate (Glucose assay kit, Sigma).

### Statistical Identification of Linear Response Phases During Perfusion

Linear regressions were performed on the temporal concentration profiles of each of the metabolites at different time-periods of the perfusion (e.g. 0–5 hrs, 0–2 hrs, etc.). This was done in order to identify whether there were multiple distinct metabolic phases during perfusion. This linear-response phase analysis served the dual purpose of establishing times to attain stability or recovery in perfusion, as well as being a necessary prerequisite to calculating fluxes accurately for metabolic flux analysis (see below for details). Box-and-whisker plots of the resulting R^2^ values for each time period and experimental group were evaluated to identify the segments of time with highest R^2^ and minimum variation (See Appendix S2 for a display of representative time periods). Ischemic livers were found to exhibit two distinct stable phases between 0–2 hrs and 2–5 hrs of perfusion. Fresh livers were found to be generally stable; greatest linearity was seen between 1–5 hrs, suggesting some degree of equilibration with the perfusate during the first hour.

Note that all metabolites displayed a generally linear trend line within the identified periods. However, for a few metabolites the concentration profiles were flat, which led to low R^2^ values despite good fit by visual inspection. These outlier R^2^ values therefore were not considered as a violation of the linearity assumption above. To avoid the analysis being influenced by such artifacts, the median and quartile analysis, which is more robust against such outliers, was employed as opposed to the mean and standard deviation.

### Calculation of Fluxes

Fluxes were calculated as the gradient of concentration of each metabolite (i.e. slope of the linear regression curve) [21] over the selected segments of perfusion, normalized to the weight of the liver and averaged for each group. Oxygen and carbon dioxide fluxes were determined every hour for each liver and were then averaged for each group over the selected segments of time.

### Data Preprocessing and Outlier Analysis

An initial outlier analysis was performed for each measurement by plotting box-and-whisker diagrams in MATLAB and eliminating obvious errors (e.g. negative values). This was followed by a more stringent analysis where for each group any measurement values above/below mean ± 2× inter-quartile range for that group were considered outliers.

### Metabolic Flux Analysis (MFA)

MFA was performed based on a stoichiometric model for the metabolic reaction network developed and tested in more detail previously [18]. The model allows for the estimation of otherwise inaccessible intracellular reaction fluxes by performing a mass balance around each intracellular metabolite using measured extracellular fluxes. The model was originally developed for perfused hypermetabolic rat livers, and this version uses a total of 28 metabolites and 62 chemical reactions, including TCA and urea cycles, amino acid metabolism, gluconeogenesis/glycolysis. The model does not incorporate complete fatty acid and lipid metabolism, and while the pentose phosphate pathway (PPP) is included, DNA synthesis/liver regeneration is assumed to be negligible. The details of these assumptions can be found elsewhere [18], [21]. The primary accommodation of the model for the gluconeogenic state is the preferential formation of oxaloacetate and phosphoenolpyruvate from pyruvate rather than acetyl-CoA, which ultimately favors gluconeogenesis. By contrast, in a glycolytic state, glycolysis is predominant and favors the formation of acetyl-CoA via pyruvate. Fluxes 6 and 7 vary therefore depending on the state of carbohydrate metabolism and are represented as dotted lines, rather than solid lines, in the glycolytic case. It is noted here that all perfused livers derived from fed rats while *in vivo* results were obtained using fasted rats. WE insulin levels were approximately 10-fold higher than what was observed in fed rats [8], [22] and without glucagon supplementation.

Briefly, in MFA the change in the concentration of intracellular metabolites is assumed to be zero (pseudo steady-state assumption) hence the sum of fluxes of each metabolite's uptake, synthesis and utilization equals zero: S.v = 0. The matrix S contains the stoichiometric coefficients of the incorporated reactions. Each element S_ij_ of S is the coefficient of metabolite i in reaction j, and each v_j_ of vector *v* is the net flux or conversion rate of reaction j.

The equation S.v = 0 is separated into measured (v_m_) and unknown fluxes (v_u_), as well as the matrices containing stoichiometric coefficients of known (S_m_) and unknown reactions (S_u_): S_u_.v_u_ = −S_m_.v_m_. The measured fluxes represent rates of uptake or release of extracellular metabolites, and by solving the equation they also give estimates of intracellular fluxes, therefore enabling an intracellular analysis based on extracellular changes. It should be noted that if the number of stoichiometric balances (i.e. independent rows, or equivalently, the rank of matrix S_u_) are equal to the number of unknown fluxes then there is a single solution. Ideally, the unknown fluxes are fewer; in this case the solution becomes a regression problem. Moreover, in this case the consistency of measured fluxes within each other and the model can be validated.

In this work, the model consistency and validity of the steady state assumption was confirmed by the method of Wang and Stephanopoulos [23]. Briefly, this approach tests if the errors form the regression for a chi-square distribution, which indicates a normal, expectable measurement error distribution. If the regression errors do not follow a chi-square distribution at p<0.05, then it is possible to identify the problematic measurements by an iterative elimination process [23] and eliminate artifactual/inconsistent measurements. This approach was used to identify two artifactual oxygen uptake measurements which, when eliminated, resolved the issues observed.

### Statistical Analysis

All statistical comparisons between individual fluxes were performed using 2-tailed Student's t-test (p<0.05).

### Groups Studied

Procured livers were either Fresh (n = 11) or exposed to an hour of warm ischemia (WI, n = 7) and then perfused, and metabolic analysis was performed. Note that it was previously demonstrated that in this model of rat DCD livers, 1 hr WI livers are not transplantable (0% survival by day 4 post transplant), however treatment by the NMP system also employed in this work increases the success to 100% (1 month post-transplant) [6]. Where necessary for comparison, *in vivo* values for rats were obtained and reported from another study [24].

## Results

We previously demonstrated that *ex vivo* normothermic perfusion of Freshly isolated rat livers results in transplantable organs, and further that the same perfusion system is necessary and sufficient for the restoration of 1 hr warm ischemic rat livers to transplantable condition [6]. We further demonstrated that the metabolic performance of the perfused rat livers are highly correlated and predictive of ischemia [8] and transplant success [25]. In this work we perform and provide additional data and analyses to assess the perfusion recovery time and organ stability. The systemic impact of perfusion on organ metabolism was evaluated through metabolic flux analysis.

### Overall Metabolic Changes due to Ischemia

**Figure 1:**
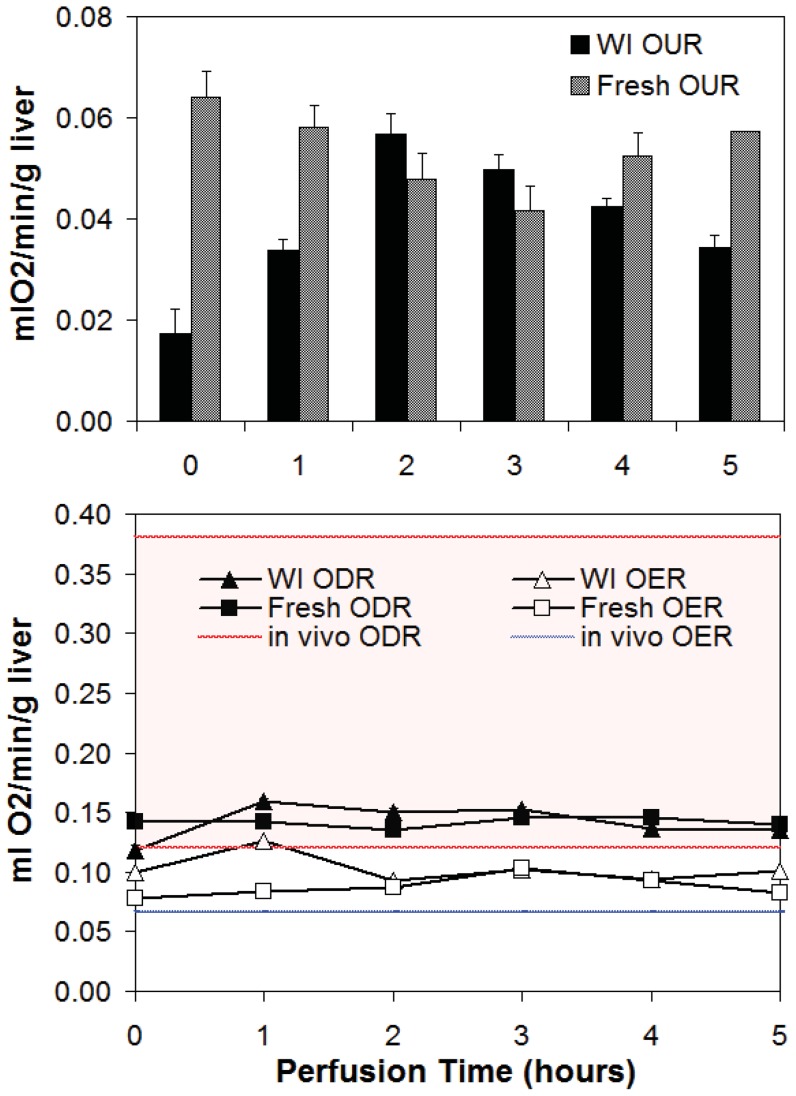
Oxygen trends in Fresh and WI livers. A) Oxygen uptake rate (OUR) for WI and Fresh livers. B) Oxygen delivery rate (ODR) and exit rate (OER) in perfused livers compared to those *in vivo*. Red and blue lines represent the range in ODR and OER (average +/− 1std dev); the range for OER is negligible.

Samples of perfusate were taken hourly at the portal vein to reflect oxygen delivery rate (ODR), and at the infra-hepatic vena cava to determine oxygen exit rate (OER). Oxygen uptake rate (OUR) was determined as the difference between ODR and OER (Figure 1A). ODR and OER were also compared to *in vivo* conditions (Figure 1B). WI livers consumed significantly less oxygen than Fresh livers during the first hour of perfusion, but by the second hour, consumption had increased to 0.050–0.057 ml O_2_/min/g liver and was comparable between groups. WI livers showed a slow but steady decline in OUR from t = 2–5 hrs, the difference between WI and Fresh livers again becoming statistically different at t = 5 hrs. Fresh livers also demonstrated a decline in OUR initially until t = 3 hrs after which consumption began to gradually increase again. Figure 1B illustrates that the ODR in perfusion averages at 0.14 ml O_2_/min/g liver in both groups, and falls within one standard deviation of the average *in vivo* ODR. The OER however, was significantly higher than *in vivo*, which has negligible variation in value, demonstrating that despite reduced oxygen supply, the liver does not consume all that is available to it in perfusion, regardless of ischemic injury.

**Figure 2:**
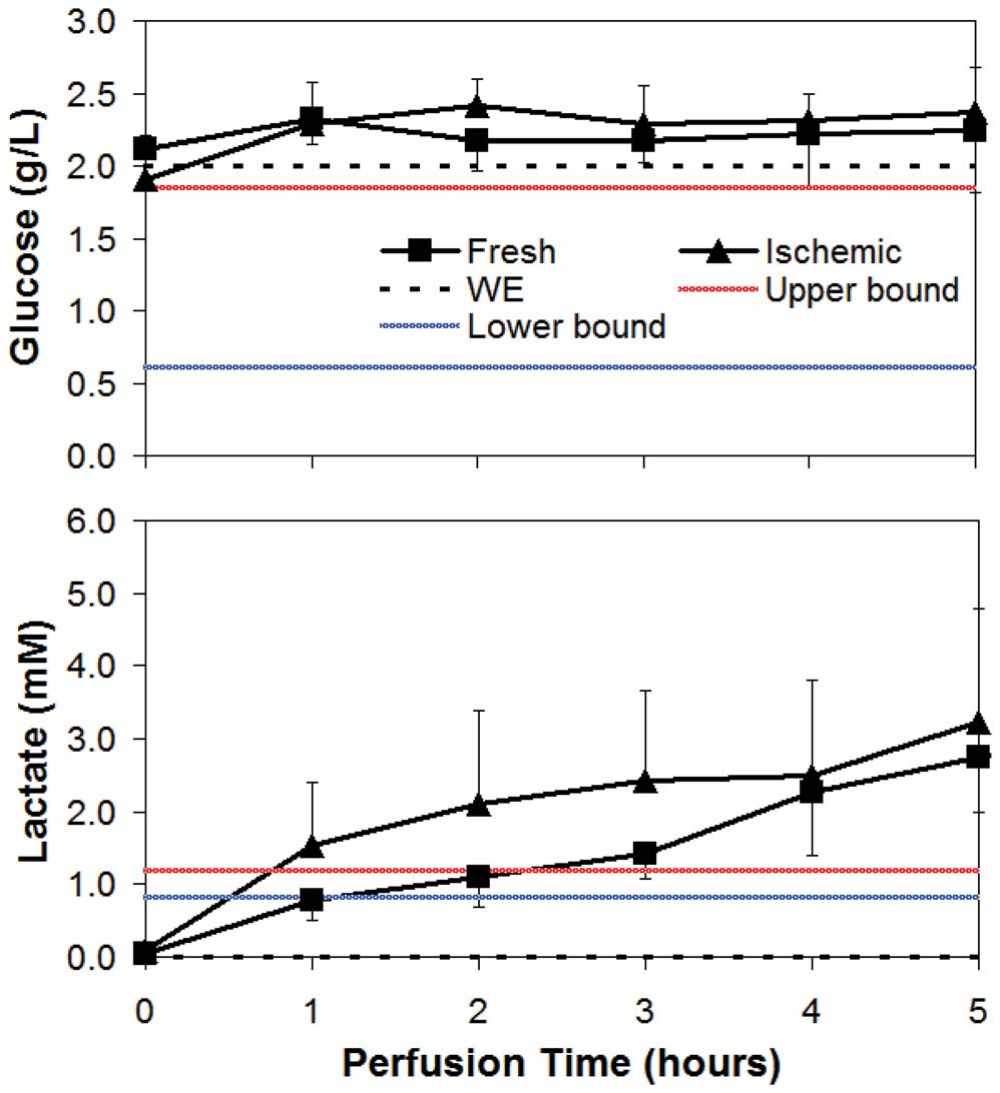
Average glucose and lactate concentrations during NELP of WI and Fresh livers. Dotted line is Williams Medium E (WE), red line is *in vivo* upper bound (ave+1 std dev) and blue is *in vivo* lower bound (ave – 1 std dev).

Concentration profiles of glucose in both WI and Fresh livers were generally stable at a value slightly above the original perfusate glucose content of 2 g/L (Figure 2A).

WI livers produced more lactate than Fresh livers, exceeding the *in vivo* upper bound value within the first hour (Figure 2B). The rate of production subsequently declined resulting in concentrations comparable to Fresh livers by the end of perfusion. Fresh livers produced lactate linearly throughout, also exceeding the *in vivo* upper limits after 2 hours of perfusion. Note that WE perfusate does not contain any lactate.

**Figure 3:**
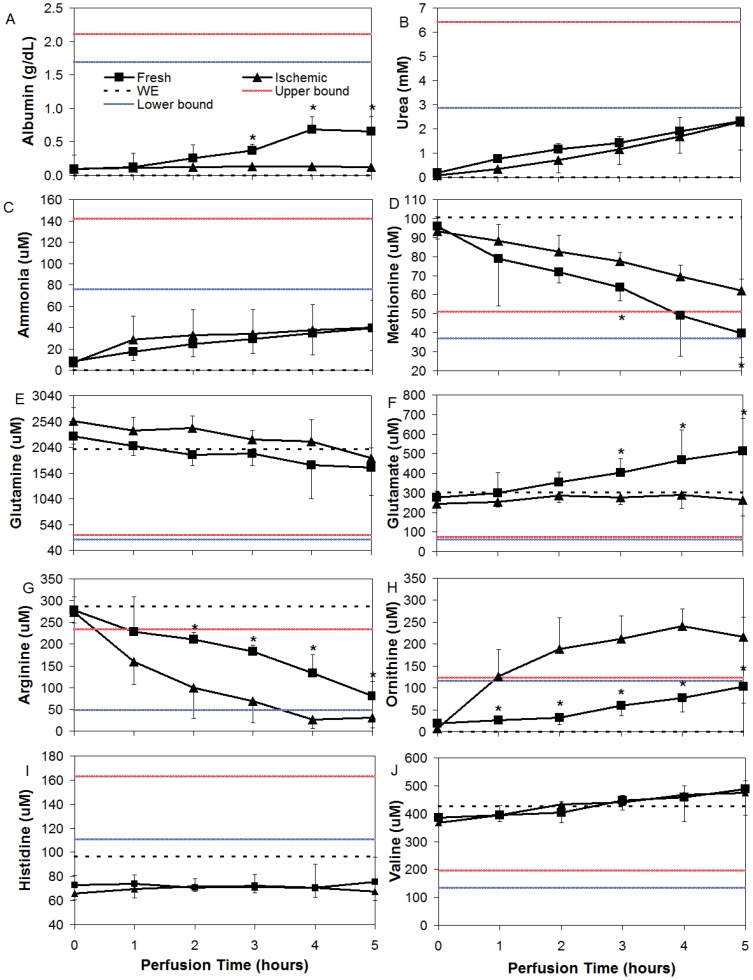
Average amino acid concentrations during NELP of WI and Fresh livers. Dotted line is Williams Medium E (WE), red line is *in vivo* upper bound (ave+1 std dev) and blue is *in vivo* lower bound (ave – 1 std dev). * Indicates significantly different from Ischemic (p<0.05).

Albumin concentration (Figure 3A) increased steadily in Fresh liver perfusions, reaching a maximum of 0.68 g/dL at t = 4 hrs, approximately 40% of the *in vivo* lower bound value. By contrast, WI livers produced little to no albumin during this time span (0.0055 g/dL/hr). Urea concentration increased similarly and linearly in both groups (Figure 3B) at a rate of 4.1 mM/hr, R^2^ = 0.99 for Fresh livers and 4.4 mM/hr, R^2^ = 0.98 for WI livers. Urea concentration also did not reach the *in vivo* lower bound concentration of approximately 2.8 mM at the end of perfusion. Ammonia concentration increased similarly in perfusate from zero to 40 uM in both groups (Figure 3C). The perfusate values of ammonia were significantly below the *in vivo* lower bound of approximately 76 uM. The amino acids methionine (Figure 3D), tyrosine, proline, lysine and phenylalanine (not shown) were all consumed at significantly lower rates in WI livers than in Fresh livers. Glutamine uptake (Figure 3E) occurred at a stable rate that was similar for both WI and Fresh livers. Rates of uptake between WI and Fresh livers were also similar for aspartate, alanine, glycine, asparagine, cysteine and threonine (not shown). WI and Fresh livers differed significantly in glutamate metabolism (Figure 3F). Glutamate concentration increased linearly in Fresh livers but was relatively unchanged in WI livers and remained within the value present in WE. Arginine (Figure 3G) by contrast was consumed at a significantly higher rate by WI livers, to the extent that it became substrate depleted at t = 4 hrs. A reciprocal increase in ornithine was observed (Figure 3H); a plateau was reached in the output by WI livers at t = 4 hrs, well above the *in vivo* upper bound value. By contrast, a linear increase in ornithine output by Fresh livers resulted in a perfusate concentration within *in vivo* range at t = 5 hrs. WE was deficient in lactate, ornithine, ammonia, urea, albumin, and ornithine at the onset of perfusion, and the liver generally increased the concentrations of each of these metabolites during perfusion. However, in the case of histidine (Figure 3I) and serine (not shown), both of which are present in WE at values significantly below the *in vivo* lower bound, neither were utilized or contributed to during perfusion. The branched chain amino acids valine (Figure 3J), isoleucine and leucine (not shown) were all produced linearly during perfusion by both groups.

### MFA of Fresh vs. Ischemic Perfused Livers

In order to identify whether there were distinct phases in liver metabolism during perfusion, linear regressions were performed on the temporal concentration profiles of each of the metabolites. Box-and-whisker plots of the resulting R^2^ values were evaluated for least variation across different segments of time (Appendix S2). Ischemic livers were found to exhibit stable but distinctly different metabolic rates between 0–2 hrs and 2–5 hrs of perfusion; MFA was conducted separately for both phases. Fresh livers were generally stable throughout perfusion; MFA was performed on the segment with greatest linearity, determined as being t = 1–5 hrs. Appendix S3 delineates the results of MFA for all groups.

**Figure 4:**
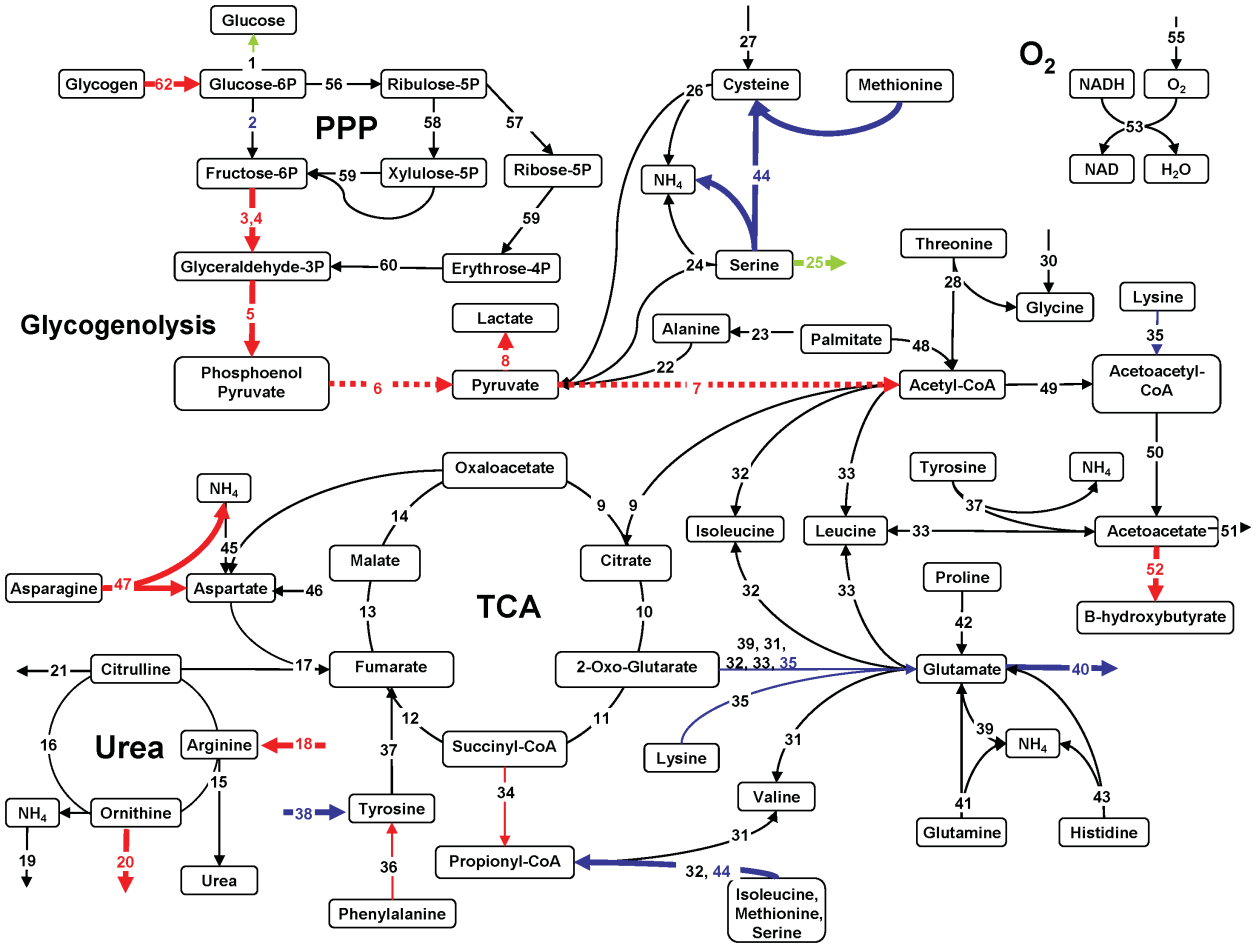
MFA of phase I (t = 0–2 hrs) for WI livers compared to Fresh livers; Fresh liver MFA forms the black baseline. Red arrows are significantly increased fluxes. Blue arrows are significantly reduced fluxes and green are reversed. Bold lines = p<0.05, Thin lines = p<0.1, Dotted lines = glycolysis.

In Figure 4, the first two hours of ischemic liver perfusion are compared to Fresh liver metabolism using MFA. The map suggests ischemic livers were significantly more glycogenolytic than Fresh livers at NMP onset, breaking down glycogen for glycolysis and glucose release. Glycolysis appeared to result in a 116% increase in the production of lactate (Flux #8). Oxygen uptake rate (Fluxes 53–55) and the TCA cycle were comparable between groups. Ischemic livers demonstrated a preferential uptake of the amino acid arginine (118% increased in Flux #18) and a reciprocal 304% increase in the release of ornithine into the extracellular space (Flux #20). Ischemic livers also showed a 46% increase in the formation of asparagine from aspartate (Flux #47). Phenylalanine uptake was increased by 24% (p<0.1, Flux #36) while tyrosine uptake was reduced by 63% (p<0.05, Flux #38) resulting in an overall reduction of fumarate production (Flux #37). Methionine and serine metabolism were significantly reduced (Flux #44); extracellular serine release was observed at this time also (Flux #25). Glutamate production (Flux #40) was 58% of that found in Fresh livers, a significant reduction due likely to a decline in contribution from lysine and 2-oxo-glutarate (Flux #35), which were reduced by 61% (p<0.1).

**Figure 5:**
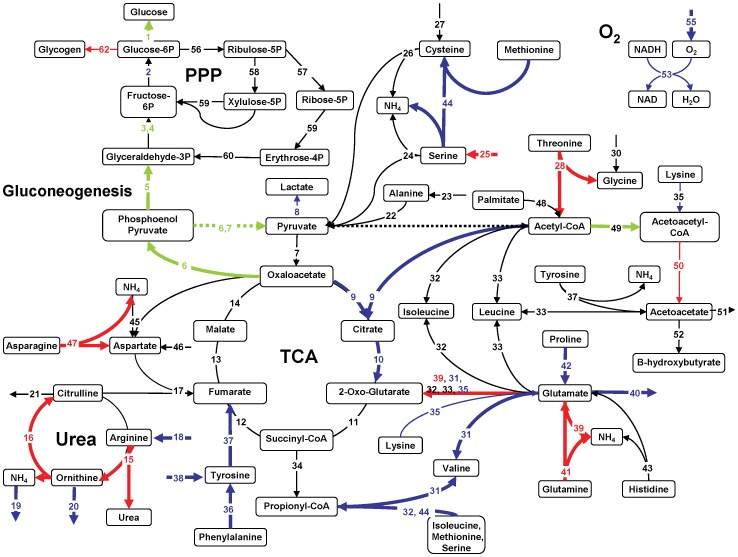
MFA of phase II (t = 3–5 hrs) for WI livers compared to Fresh livers; Fresh liver MFA forms the black baseline. Red arrows are significantly increased fluxes. Blue arrows are significantly reduced fluxes and green are reversed. Bold lines = p<0.05, Thin lines = p<0.1, Dotted lines = glycolysis.

Between 2–5 hrs of WI perfusion (Figure 5) more differences were apparent between Fresh and WI livers than at 0–2 hrs. WI livers appeared to be mildly gluconeogenic and demonstrated a 30% reduction in lactate output compared to Fresh livers, despite a further decline in oxygen uptake rate. Contributions to the TCA cycle via phenylalanine conversion to tyrosine were reduced, such that fumarate production via this pathway (Flux #37) was only 50% of Fresh liver flux values. Reduced acetyl-CoA and oxaloacetate (Flux #9) resulted in a 61% reduction of citrate formation, while threonine conversion to acetyl-CoA was increased 470%. Glutamate output was further reduced to within 4% of Fresh liver fluxes; contributions to its formation from both lysine (50% of Fresh livers, Flux #35) and proline (40% of Fresh livers, Flux #42) impacted its production substantially. There was however, a 260% increase in glutamate formation via glutamine (Flux #41), which resulted in a 100% increase in glutamate formation of 2-oxo-glutarate (Flux #39). This increased flux converged on the TCA cycle at a point of reduced incoming fluxes from citrate, such that the downstream pathway of the cycle was restored to a value similar to that of Fresh livers. Asparagine to aspartate production was further increased to a rate 60% greater than Fresh livers, while tyrosine, methionine and serine metabolism remained reduced, though extracellular serine was then actively consumed at a higher rate (Flux #25). Arginine uptake and ornithine output were significantly reduced compared to Fresh livers, but the increased metabolism of asparagine, threonine and glutamine resulted in an overall increased urea cycle.

**Figure 6:**
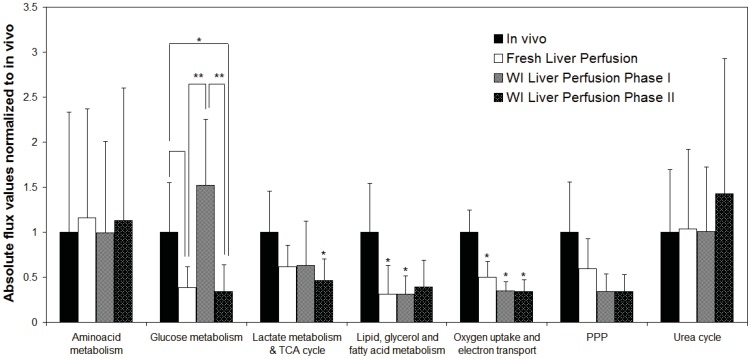
Summary of major pathways in perfused livers normalized to *in vivo* values.

Figure 6 summarizes the MFA findings demonstrating that perfused liver activity of the major pathways of metabolism including oxygen uptake, electron transport, lipid oxidation, the TCA cycle and the PPP was reduced compared to *in vivo* values [24]. Lactate production was increased in perfusion while amino acids and the urea cycle were similar to *in vivo* livers. All perfused livers released branched chain amino acids and had negligible histidine uptake compared to *in vivo* livers. Perfused livers were generally glycolytic compared to fasted gluconeogenic *in vivo* livers, though extracellular glucose content varied little in concentration during perfusion.

## Discussion

Perfusion systems are capable of significantly impacting the availability of transplantable organs by optimally supporting donor organs during storage, and recovering reversibly damaged tissues through perfusate-based treatment protocols [26], [27]. To facilitate the translation of perfusion technology to clinical use, comprehensive and dynamic analyses of organ function during perfusion are needed that identify parameters critical to organ stability and recovery. As a first step in this direction, we performed a comprehensive metabolic analysis to capture the time of ischemic liver recovery and evaluate the impact of perfusate on organ stability. We conducted our study on hourly perfusate samples from Fresh and WI livers that were successfully transplanted after 6 hours of preservation. Through the measurement of 28 metabolites and the calculation of an additional 34 fluxes using MFA, we were able to evaluate the stability of our perfusion system, identify perfusate short-comings, and establish significant differences between Fresh and WI livers useful in the future design of treatment protocols.

Fresh livers highlighted baseline factors to be considered in future perfusions. They demonstrated functional stability through linear changes in metabolite concentrations during perfusion; greatest stability/linearity was seen after the first hour of perfusion, likely reflecting a period of adjustment to the *ex vivo* environment. There was no evidence of substrate limitation in the perfusate at t = 6 hours. MFA depicted the expected response to a fed/high insulin state by showing glucose uptake, glycogen storage and glycolysis. Alanine and lactate uptake, the usual precursors for gluconeogenesis were down-regulated and produced, respectively. The TCA cycle was reduced, as were ketone body production and fatty acid oxidation, altogether requiring a minimum of oxygen, concomitant with a high energy state.

Restoration of ischemic livers during perfusion appears to be correlated with a distinct change in liver metabolism at t = 2 hrs as seen using linear regression analysis on the temporal concentration profiles of each of the metabolites. The restoration of oxygen uptake rates to Fresh liver values at t = 2 hrs, followed by a relative plateau in oxygen consumption was the most dramatic change amongst the metabolites measured and coincides with Pastor's [28] description that this marks the full recovery of the organ from ischemic damage. It is interesting to note that in MP applications typically longer perfusion durations are chosen [4], [6] whereas these results suggest a much shorter time to transplantation may be sufficient. It is further possible that optimizing the delivery of oxygen during perfusion may result in even shorter ischemic organ recovery times [28], as discussed below.

The value of optimal oxygen delivery, in that it is delivered to all cells at an acceptable rate, is appreciated upon closer inspection of the data. From *in vivo* data [24] it can be deduced that a healthy rat liver consumes approximately 0.25 ml O_2_/min/g liver in the fasted state. Rearranging the abovementioned equation to calculate oxygen concentration, using a pO_2_ of approximately 500 mHg in perfusion, and assuming a 90% oxygenation of the hemoglobin present, the maximum value of hemoglobin the liver needs to ensure sufficient oxygenation at physiological flow rates and portal pressures is approximately 8 g/dL or 23%. Riedel et. al. [29] verified experimentally that the optimal hematocrit in perfusion was approximately 20%, above which oxygen uptake rates did not increase with flow rates while portal pressures became destructively high. Similarly, we found that in order to sustain a physiological perfusion pressure in our system, adjustment of the hematocrit through dialysis was optimal at values between 15–20%. Nevertheless, despite meeting the oxygen requirements of the liver, perfused livers did not take up all the oxygen available to them, suggesting an overall damped rate of metabolic activity. Several publications concur with our findings of lower-than-physiological oxygen uptake rates [3], [15], [30] to the extent that Mischinger et. al. [13] favor removing erythrocytes altogether, resulting in a significantly simpler perfusion setup. Before this can be done however, it is necessary to determine why the livers are not consuming all the available oxygen.

It is possible that livers in perfusion are functionally incapable of maximizing oxygen extraction. There may be several mechanisms potentially involved in this response: The first, alluded to in the discussion on Fresh livers above, may simply be that livers in perfusion do not need to be as metabolically active in their non-demanding *ex vivo* environment. Alternatively, the delivery of perfusate oxygen at the lowest range of typical *in vivo* values for prolonged periods of time (hours) (Figure 1) may be resulting in a systemic hypoxia-induced mitochondrial inhibition [31]. Third, the metabolic consequences of ischemia reperfusion injury may be causing localized areas of hypoperfusion in the microvasculature [32], [33].

Several factors have been implicated in the metabolic response to hypoxia, which serve to minimize organ dependence on oxygen by reducing anabolic pathways and increasing catabolic pathways, using anaerobic respiration as the end-point:

Hypoxia-inducible factor (HIF) [31], as the name suggests, is rapidly incurred during periods of low or insufficient oxygen. HIF strongly induces glycolysis, also referred to as the Pasteur effect [34], as seen dominantly in Figure 4, Fluxes #2–6. HIF is also responsible for the preferential formation of lactate from pyruvate (Flux #8) by upregulation of lactate dehydrogenase. This serves to enable the production of ATP, while decreasing the contribution of acetyl-CoA to the TCA cycle (Flux #9), effectively inhibiting it [35] (Fluxes #9–14), thereby reducing cellular dependence on oxygen. Also, HIF induces pyruvate dehydrogenase (PDH) kinase-1 that phosphorylates and inactivates PDH, the mitochondrial enzyme that converts pyruvate into acetyl-CoA. Our findings do not suggest that the rate of formation of acetyl-CoA from pyruvate is impeded by warm ischemia, however in all perfused livers, the TCA cycle is reduced compared to *in vivo*. Therefore, the results of MFA generally agree with these expected effects of HIF, suggesting the persistence of hypoxia in both ischemic and Fresh livers.

AMP-activated protein kinase (AMPK) is also stimulated during hypoxia and ischemia, and functions to suppress the overall metabolic rate and hence energy requirements of cells for self-preservation [36]. AMPK is associated with increasing glycolysis and minimizing gluconeogenesis [37] thereby largely coinciding with the effects of HIF and the MFA findings. It is also associated with increasing lipid oxidation and decreasing fat storage, notably opposing many insulin pathways [38], [39], which is not observed in the MFA. The preferential reduction in palmitate oxidation (Flux #48) seen in Figures 4–6 illustrates the likely domination of a high insulin state over AMPK, controlling lipid metabolism. It is unclear at this point whether promoting the reduction of lipid oxidation through higher insulin values is an effective means of reducing the damaging consequences of lipid peroxidation at the expense of reduced ATP production; subsequently further optimization of perfusate insulin and fatty acid levels is necessary.

Nitric oxide (NO) has been identified as another important determinant of metabolism during hypoxia, facilitating the distribution of available oxygen both at the mitochondrial and vascular scale [40], [41]. In the mitochondrion, there is an increased NO accumulation at low O_2_ levels, when cytochrome c oxidase is primarily reduced. NO actively competes with the enzyme and suppresses the respiratory rate, which coincides with the effects of HIF [40], [41], [42], [43], [44], [45]. Increased intracellular NO has also been shown to reduce protein synthesis, which requires energy expenditure, during hypoxia [36], in particular inhibiting the formation of albumin, the effects of which may last for several hours after NO exposure [46]. From our findings, albumin synthesis in WI livers remained flat for the duration of perfusion (Figure 3), while Fresh livers began to recover by t = 2 hours, logically suggesting they had less NO exposure. At the vascular level, the accumulation of NO intracellularly results in reduced NO secretion, and subsequent vasoconstriction that serve to extend the ischemic time despite initiation of reperfusion [47]. Alteration of NO production is also supported by the comparatively increased extracellular arginine uptake seen in WI livers, with a reciprocal increase in ornithine production. Arginine is a precursor for NO, but the balance between arginases and nitric oxide synthases are dramatically altered during hypoxia [42], [48], [49] possibly favoring an ineffective shuttling of arginine to ornithine as opposed to the desirable production of NO.

Given the importance of adequate oxygenation during perfusion, one possible consideration is to reduce the liver's dependence on oxygen even further by reducing the perfusate temperature, which has also been associated with less post-ischemic vasoconstriction [50]. We [11], [51] and others [52] have shown that room temperature perfusion significantly reduces whole organ metabolism but still results in viable, transplantable organs. This could also ensure that use of erythrocytes as oxygen carriers is not necessary. The combination of erythrocyte removal and room temperature perfusion would simplify the machine perfusion approach substantially, addressing a major factor in the reluctance to utilize machine perfusion systems clinically. Further measures to ensure patent microvasculature [12] and desirable flow rates would be the addition of thrombolytics, edema-reducing colloids or impermeants, and vasodilators, including arginine [53], [54], [55].

The reduced metabolic demand on the *ex vivo* liver effectively minimizes the perfusate requirements to keep the liver alive and assess its function. Amino acids may offer the greatest room for further perfusate optimization. Their metabolism in perfused livers helpfully reflected the livers' abilities to restore perfusate content to physiological levels and utilize particular amino acids for specific applications, such as the vasodilatation function of arginine. Fisher and Kerly [56], who performed analyses of amino acid metabolism in healthy fasted perfused rat livers, also observed a steady increase in perfusate content of branched chain amino acids (BCAAs) valine, isoleucine and leucine, as seen in both the Fresh and WI livers in this work. Further, these authors found both histidine and glutamate to be unchanged in perfusion; both trends held true in WI livers. Fresh livers demonstrated a net production of glutamate, which may be related to a normal transamination of excessive perfusate amino acids in conjunction with an otherwise reduced TCA cycle. Together these findings support a high degree of substrate specificity by the liver in perfusion and a regulated response to supraphysiological perfusate content. A reduction in the concentration of some amino acids, including the BCAAs, histidine and glutamate, may serve to further reduce the metabolic activity of the liver by reducing urea cycle activity.

## Conclusions

In summary, this comprehensive metabolic analysis of the performance of Fresh and WI livers in normothermic MP demonstrates sustained organ stability over 5–6 hours of perfusion, and the restoration of WI livers to a likely transplantable state within 2 hours of perfusion.

The data presented in this study provide the bases for rational MP optimization. In particular, lipid oxidation was suppressed, likely due to the high insulin levels, while amino acids were extensively metabolized, likely due to supraphysiological perfusate concentrations. Perfused livers did not consume all the available oxygen and were hypoxic independent of ischemic injury suggesting that enhanced microcirculation via vasodilators and anti-thrombolytics might be an effective approach at optimizing the delivery of oxygen to hepatocytes in both groups as early in perfusion as possible.

Further, the data and analyses presented here enable the conduct of future meta-analyses, such as Flux Balance Analysis [57], [58], [59], [60], which are necessary steps in the building of automated feedback control systems for real-time metrics of organ viability and support [61], [62].

## Supporting Information

Appendix S1 **In vivo and Perfusate (WE) Reference Concentrations.**
(DOCX)

Appendix S2 **Box-and-whisker plots of linear regressions performed on the temporal concentration profiles of 28 metabolites measured for WI and Fresh livers.**
(DOCX)

Appendix S3 **Metabolic Flux Analysis (µmol/hr/g liver).**
(DOCX)
