# Supplementary material for: Resuscitation of Ischemic Donor Livers with Normothermic Machine Perfusion: A Metabolic Flux Analysis of Treatment in Rats
Source: PLoS One. 2013 Jul 26;8(7):e69758. doi: 10.1371/journal.pone.0069758 (PMC3724866; doi:10.1371/journal.pone.0069758)
Supplement: Appendix S1 — In vivo and Perfusate (WE) Reference Concentrations. (DOCX) [file pone.0069758.s001.docx]

**Appendix A.** In vivo and Perfusate (WE) Reference Concentrations

| Metabolite | Portal Vein^1^ | Williams Medium E^2^ |
| --- | --- | --- |
| Acetoacetic acid (μM) | 110±73 | 0 |
| Alanine (μM) | 397±13 | 1010 |
| Albumin (g/dL) | 1.9±0.21 | 0 |
| Ammonia (μM) | 109±33 | 0 |
| Arginine (μM) | 141±93 | 287 |
| Asparagine (μM) | 49±5.4 | 151 |
| Aspartate (μM) | 21±7.6 | 225 |
| b-Hydroxybutyric acid (μM) | 110±96 | 0 |
| Cysteine (μM) | 15±1.5 | 330 |
| Glucose (g/dL) | 123±62 | 0 |
| Glutamate (μM) | 68±7.7 | 302 |
| Glutamine (μM) | 293±46 | 2000 |
| Glycine (μM) | 273±27 | 666 |
| Histidine (μM) | 137±26 | 97 |
| Isoleucine (μM) | 86±16 | 381 |
| Lactate (mM) | 1.0±0.18 | 0 |
| Leucine (μM) | 261±33 | 572 |
| Lysine (μM) | 220±38 | 598 |
| Methionine (μM) | 44±7.0 | 101 |
| Ornithine (μM) | 120±3.5 | 0 |
| Phenylalanine (μM) | 59±3.9 | 151 |
| Proline (μM) | 163±12 | 261 |
| Serine (μM) | 199±13 | 95 |
| Threonine (μM) | 204±41 | 336 |
| Tyrosine (μM) | 68±7.4 | 278 |
| Urea nitrogen (mM) | 4.6±1.8 | 0 |
| Valine (μM) | 165±31 | 427 |

^1^ Izamis ML, et. al. In situ metabolic flux analysis to quantify the liver metabolic response to experimental burn injury, 2011.

^2^ Sigma-Aldrich cat. #W1878
