# Supplementary material for: Resuscitation of Ischemic Donor Livers with Normothermic Machine Perfusion: A Metabolic Flux Analysis of Treatment in Rats
Source: PLoS One. 2013 Jul 26;8(7):e69758. doi: 10.1371/journal.pone.0069758 (PMC3724866; doi:10.1371/journal.pone.0069758)
Supplement: Appendix S2 — Box-and-whisker plots of linear regressions performed on the temporal concentration profiles of 28 metabolites measured for WI and Fresh livers. (DOCX) [file pone.0069758.s002.docx]

**Appendix B:** Box-and-whisker plots of linear regressions performed on the temporal concentration profiles of 28 metabolites measured for WI and Fresh livers.


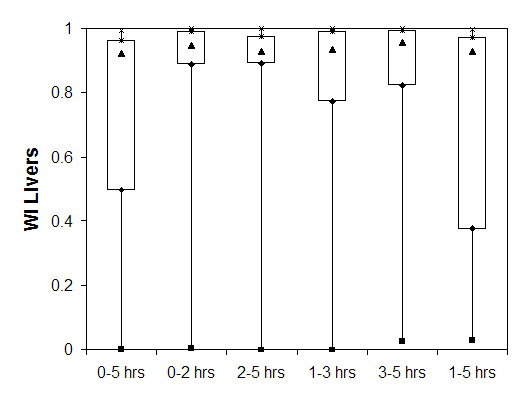


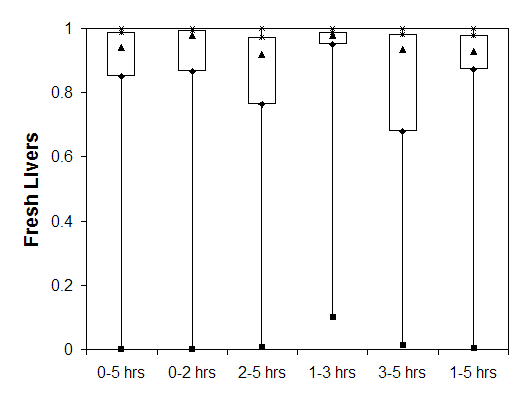


Legend: ■ Minimum. ♦ First quartile. ▲ Median. * Third quartile. × Maximum.
