# Supplementary material for: Resuscitation of Ischemic Donor Livers with Normothermic Machine Perfusion: A Metabolic Flux Analysis of Treatment in Rats
Source: PLoS One. 2013 Jul 26;8(7):e69758. doi: 10.1371/journal.pone.0069758 (PMC3724866; doi:10.1371/journal.pone.0069758)
Supplement: Appendix S3 — Metabolic Flux Analysis (µmol/hr/g liver). (DOCX) [file pone.0069758.s003.docx]

**Appendix C. Metabolic Flux Analysis (µmol/hr/g liver)**

| # | REACTION | PATHWAY | FRESH: T=1-5hr | WI: T=0-2hr | WI: T=2-5hr |
| --- | --- | --- | --- | --- | --- |
| 1 | Glucose 6-phosphate ↔ Glucose | Gluconeogenesis | -31±15 | 88±201 | **44±17** |
| 2 | Fructose 6-phosphate ↔ Glucose 6-phosphate | Gluconeogenesis | 24±85 | -26±71 | 22±25 |
| 3 | Fructose 1,6-Bisphosphate ↔ Fructose6-phosphate | Gluconeogenesis | -5±21 | **-43±51** | 6±8^†^ |
| 4 | 2 Glyceraldehyde 3-P ↔ Fructose 1,6-Bisphosphate | Gluconeogenesis | -5±21 | **-43±51** | 6±8^†^ |
| 5 | Phosphoenolpyruvate + NADH ↔Glyceraldehyde 3-P | Gluconeogenesis | -24±26 | **-94±99** | **4±14**^†^ |
| 6 | Oxaloacetate ↔ CO_2_ +Phosphoenolpyruvate (Gluconeogenic) Phosphoenolpyruvate + ADP → Pyruvate (Glycolytic) | Gluconeogenesis | -24±26 | **-94±99** | **4±14**^†^ |
| 7 | Pyruvate + CO_2_ ↔ Oxaloacetate (Gluconeogenic) Pyruvate + CoA + NAD+ → Acetyl-CoA + CO_2_ + NADH (Glycolytic) | Gluconeogenesis | -37±23 | **-110±101** | -24±12^†^ |
| 8 | Lactate ↔ Pyruvate + NADH | Lactate metabolism & TCA cycle | -57±21 | **-124±98** | -40±12^†^ |
| 9 | Acetyl-CoA + Oxaloacetate →Citrate | Lactate metabolism & TCA cycle | 31±14 | 20±26 | **12±9** |
| 10 | Citrate ↔ 2-oxo-Glutarate + NADH + CO_2_ | Lactate metabolism & TCA cycle | 31±14 | 20±26 | **12±9** |
| 11 | 2-oxo-Glutarate → Succinyl-CoA + NADH + CO_2_ | Lactate metabolism & TCA cycle | 35±14 | 30±26 | 34±9 |
| 12 | Succinyl-CoA ↔ FADH2 +Fumarate | Lactate metabolism & TCA cycle | 32±14 | 26±26 | 32±9 |
| 13 | Fumarate ↔ Malate | Lactate metabolism & TCA cycle | 69±19 | 56±27 | 56±17 |
| 14 | Malate ↔ Oxaloacetate + NADH | Lactate metabolism & TCA cycle | 69±19 | 56±27 | 56±17 |
| 15 | Arginine → Ornithine + Urea | Urea cycle | 35±12 | 35±4 | **57±4**^†^ |
| 16 | Ornithine + CO_2_ + NH_4_ ↔Citrulline | Urea cycle | 31±10 | 25±8 | **56±4**^†^ |
| 17 | Citrulline + Aspartate →Arginine + Fumarate | Urea cycle | 29±10 | 25±8 | 20±13 |
| 18 | Arginine uptake | Aminoacid metabolism | 4.3±1.6 | **9.3±6.7** | 2.6±0.6^†^ |
| 19 | Ammonia Output | Urea cycle | 0.7±0.2 | 0.8±0.9 | **0.5±0.1** |
| 20 | Ornithine Output | Urea cycle | 2.3±1.2 | **9.3±6** | **0.7±1.1**^†^ |
| 21 | Citrulline Output | Aminoacid metabolism | 2±1.9 | -0.1±9 | 2±1.2 |
| 22 | Alanine → Pyruvate + NH_4_ + NADH | Aminoacid metabolism | 8.6±2.4 | 4.9±8.3 | 7.2±1.8 |
| 23 | Alanine Output | Aminoacid metabolism | -7.1±1.4 | -3.9±7.6 | -7.2±1.8 |
| 24 | Serine → Pyruvate + NH_4_ | Aminoacid metabolism | 8.6±6.3 | 6.7±13.5 | 6.6±1.5 |
| 25 | Serine Uptake | Aminoacid metabolism | 0.2±0.3 | **-1.2±1.1** | **0.5±0.2**^†^ |
| 26 | Cysteine → Pyruvate+ NH_4_ + NADH | Aminoacid metabolism | 2.9±3.2 | 3±4.8 | 2.1±0.5 |
| 27 | Cysteine Output | Aminoacid metabolism | -0.1±2.7 | -1.4±1.9 | -1.2±0.5 |
| 28 | Threonine → NADH + Glycine +Acetyl-CoA | Aminoacid metabolism | 0.4±0.7 | 0.6±8.2 | **2.1±1.2** |
| 29 | Glycine ↔ CO_2_ + NH_4_ +NADH | Aminoacid metabolism | 8.2±4.2 | 7.5±10.2 | 6.9±1.4 |
| 30 | Glycine Uptake | Aminoacid metabolism | 4.8±0.9 | 5±0.1 | 4.8±0.8 |
| 31 | Valine + 2-oxo-Glutarate → Glutamate + propionyl-CoA + 3 NADH +FADH2 + 2 CO_2_ | Aminoacid metabolism | -2.2±0.9 | -2.9±1 | **-0.9±1.4**^†^ |
| 32 | Isoleucine + 2-oxo-Glutarate → Glutamate + propionyl-CoA + Acetyl-CoA + 2 NADH + FADH2 +CO_2_ | Aminoacid metabolism | -1.7±0.6 | -1.4±0.3 | -1.7±0.7 |
| 33 | Leucine + 2-oxo-Glutarate → Glutamate + NADH + FADH2 Acetoacetate + Acetyl-CoA | Aminoacid metabolism | -2.6±2.1 | -4±3.5 | -2.4±1 |
| 34 | Propionyl-CoA + CO_2_ ->Succinyl-CoA | Aminoacid metabolism | -2.6±1.1 | -3.7±1 | -1.7±1.6^†^ |
| 35 | Lysine + 2 2-oxo-Glutarate → 2 Glutamate + 4 NADH + FADH2 + 2CO2 + Acetoacetatyl-CoA | Aminoacid metabolism | 7.2±4.2 | 2.9±6 | 3.7±1.6 |
| 36 | Phenylalanine + O_2_ → Tyrosine | Aminoacid metabolism | 2.1±0.6 | 2.6±0.4 | **0.9±0.2**^†^ |
| 37 | Tyrosine + 2 O_2_ →NH4 + CO_2_ + Fumarate + Acetoacetate + NADH | Aminoacid metabolism | 7.5±2.2 | 5±4.8 | **3.6±0.2** |
| 38 | Tyrosine Output | Aminoacid metabolism | -3.9±0.4 | **-1.5±2** | **-2.7±0.1** |
| 39 | Glutamate ↔ 2-oxo-Glutarate + NADH +NH_4_ | Aminoacid metabolism | 12.1±9.4 | 7.2±24.9 | **24.2±6.8** |
| 40 | Glutamate Output | Aminoacid metabolism | 5.4±3.5 | **2.3±0.1** | **0.2±0.9**^†^ |
| 41 | Glutamine → Glutamate + NH_4_ | Aminoacid metabolism | 5.7±3.3 | 9.3±25.9 | **20.4±5.6** |
| 42 | Proline +0.5 O_2_→ Glutamate + 0.5 NADH | Aminoacid metabolism | 2.6±0.4 | 2±1.9 | **1.6±0.9** |
| 43 | Histidine → NH_4_ +Glutamate | Aminoacid metabolism | -0.2±0.4 | -0.2±0.3 | 0.1±0.2 |
| 44 | Methionine + Serine → Cysteine + NADH + Propionyl-CoA + CO2 | Aminoacid metabolism | 1.3±0.2 | **0.6±0.1** | **0.9±0.2**^†^ |
| 45 | Aspartate↔Oxaloacetate + NH_4_ +NADH | Aminoacid metabolism | -24±8.7 | -20.3±10.9 | -15.9±12.9 |
| 46 | Aspartate Uptake | Aminoacid metabolism | 2.5±0.7 | 2.1±1.9 | 2.6±0.1 |
| 47 | Asparagine → Aspartate + NH_4_ | Aminoacid metabolism | 0.8±0.4 | **1.2±0** | **1.4±0.3** |
| 48 | Palmitate→ 8 Acetyl-CoA +7 FADH2 + 7 NADH | Lipid, glycerol, fatty acid metabolism | 4.2±2.3 | 4.1±4 | 3.2±1.3 |
| 49 | 2 Acetyl-CoA ↔ Acetoacetyl-CoA | Lipid, glycerol, fatty acid metabolism | -0.8±4.9 | 3.9±14.1 | **5.5±2.1** |
| 50 | Acetoacetyl-CoA → Acetoacetate | Lipid, glycerol, fatty acid metabolism | 6.5±3.5 | 6.7±13.2 | 9.2±1.3 |
| 51 | Acetoacetate Output | Lipid, glycerol, fatty acid metabolism | 11.4±1.8 | 7.7±11.9 | 10.4±0.9 |
| 52 | Acetoacetate + NADH ↔b-Hydroxybutyrate | Lipid, glycerol, fatty acid metabolism | 0±0 | **0±0** | 0±0 |
| 53 | NADH + 0.5 O_2_ → NAD | Oxygen uptake and electron transport | 164±52 | 109±87 | 119±32 |
| 54 | FADH2 + 0.5 O_2_ → FAD | Oxygen uptake and electron transport | 62±26 | 49±44 | 53±16 |
| 55 | O2 Uptake | Oxygen uptake and electron transport | 132±38 | 92±63 | **95±24** |
| 56 | Glucose 6-phosphate→ 2 NADPH + CO_2_ +Ribulose 5-P | PPP | 43±100 | 25±61 | 25±29 |
| 57 | Ribulose 5-P ↔ Ribose 5-P | PPP | 14±33 | 8±20 | 8±10 |
| 58 | Ribulose 5-P ↔ Xylulose 5-P | PPP | 29±67 | 17±40 | 16±19 |
| 59 | Ribose 5-P + Xylulose 5-P ↔ Fructose6-P + Erythrose 4-P | PPP | 14±33 | 8±20 | 8±10 |
| 60 | Erythrose 4-P + Xylulose 5-P ↔ Glyceraldehyde 3-P + Fructose 6-P | PPP | 14±33 | 8±20 | 8±10 |
| 61 | CO_2_ Output | Oxygen uptake and electron transport | 120±97 | 81±39 | **60±23** |
| 62 | Glycogen ↔ Glucose-6-P | Glucose metabolism | -13±26 | 139±208 | -34±14^†^ |

**Bolded** items are significantly different (p<0.05) from FRESH.

^†^ Items significantly different from ISCHEMIC T=0-2hrs
